# Initial experience with an electron FLASH research extension (FLEX) for the Clinac system

**DOI:** 10.1002/acm2.14159

**Published:** 2023-09-21

**Authors:** Kyuhak Oh, Kyle J. Gallagher, Megan Hyun, Diane Schott, Sarah Wisnoskie, Yu Lei, Samuel Hendley, Jeffrey Wong, Shuo Wang, Brendan Graff, Christopher Jenkins, Frank Rutar, Md Ahmed, Joshua McNeur, Jeffrey Taylor, Marty Schmidt, Lasitha Senadheera, Wendy Smith, Donald Umstadter, Subodh M. Lele, Ran Dai, Dong Jianghu (James), Ying Yan, Zhou Su‐min

**Affiliations:** ^1^ University of Nebraska Medical Center Omaha Nebraska USA; ^2^ Varian Medical Systems Palo Alto California USA; ^3^ University of Nebraska Lincoln Nebraska USA

**Keywords:** cell viability, conventional dose rate (CONV), dosimetry, electron Ultra‐high dose rate (eFLASH), FLASH Research Extension (FLEX), linear accelerator, stress‐activated senescence

## Abstract

**Purpose:**

Radiotherapy delivered at ultra‐high‐dose‐rates (≥40 Gy/s), that is, FLASH, has the potential to effectively widen the therapeutic window and considerably improve the care of cancer patients. The underlying mechanism of the FLASH effect is not well understood, and commercial systems capable of delivering such dose rates are scarce. The purpose of this study was to perform the initial acceptance and commissioning tests of an electron FLASH research product for preclinical studies.

**Methods:**

A linear accelerator (Clinac 23EX) was modified to include a non‐clinical FLASH research extension (the Clinac‐FLEX system) by Varian, a Siemens Healthineers company (Palo Alto, CA) capable of delivering a 16 MeV electron beam with FLASH and conventional dose rates. The acceptance, commissioning, and dosimetric characterization of the FLEX system was performed using radiochromic film, optically stimulated luminescent dosimeters, and a plane‐parallel ionization chamber. A radiation survey was conducted for which the shielding of the pre‐existing vault was deemed sufficient.

**Results:**

The Clinac‐FLEX system is capable of delivering a 16 MeV electron FLASH beam of approximately 1 Gy/pulse at isocenter and reached a maximum dose rate >3.8 Gy/pulse near the upper accessory mount on the linac gantry. The percent depth dose curves of the 16 MeV FLASH and conventional modes for the 10 × 10 cm^2^ applicator agreed within 0.5 mm at a range of 50% of the maximum dose. Their respective profiles agreed well in terms of flatness but deviated for field sizes >10 × 10 cm^2^. The output stability of the FLASH system exhibited a dose deviation of <1%. Preliminary cell studies showed that the FLASH dose rate (180 Gy/s) had much less impact on the cell morphology of 76N breast normal cells compared to the non‐FLASH dose rate (18 Gy/s), which induced large‐size cells.

**Conclusion:**

Our studies characterized the non‐clinical Clinac‐FLEX system as a viable solution to conduct FLASH research that could substantially increase access to ultra‐high‐dose‐rate capabilities for scientists.

## 1 INTRODUCTION

Radiotherapy delivered with ultra‐high‐dose‐rates (>40 Gy/s), known as FLASH, has the potential to change the landscape of radiotherapy and improve the care of cancer patients. The concept was first mentioned by Dewey and Boag^1^ in 1959 and reignited by Favaudon et al.^2^ in 2014. In their seminal work, Favaudon et al. demonstrated the potential for using FLASH radiation to efficiently eliminate cancer cells while sparing healthy organs and tissues, thus effectively widening the therapeutic window. Since then, there has been a significant amount of research focused on FLASH radiotherapy, including beam commissioning,^3^, ^4^ dosimetry,^5^ small animal experiments,^6^, ^7^ and preclinical trials.^3^, ^8^ In 2018, the first clinical implementation of electron FLASH radiotherapy was successfully conducted for the treatment of a patient with a skin lesion of recurrent cutaneous lymphoma.^9^ More recently, Mascia et al.^10^ reported the initial results from the first clinical trial, that is, FAST‐01, and have demonstrated the safe use of proton FLASH radiotherapy for the palliation of patients with bone metastasis. However, the mechanism for the FLASH effect remains largely unknown, and further research is warranted for successful clinical implementation.^11^

While the FLASH effect may promise great potential to improve radiotherapy for cancer patients, the biological mechanisms underlying the effect remain poorly understood. This gap in knowledge is partly attributed to the scarcity of accessible and affordable FLASH radiation delivery systems for biomedical researchers. Currently, the linear accelerator (linac) is the workhorse of radiation oncology and is the world's most commonly used radiotherapy delivery system.^12^ Therefore, the capacity for FLASH research could be substantially increased if conventional linacs were modified into FLASH‐capable delivery systems.

There are several challenges in converting a conventional linac to be capable of producing FLASH dose rates. In particular, there is a vast gap in radiation production efficiency between the photon and electron modes on a linac due to the low conversion rate of the incident electrons impinging on the target to generate bremsstrahlung photons. Thus, it is easier to achieve FLASH dose rates by utilizing the high throughput photon mode without the x‐ray target and, additionally, employing little to no scattering foil to spread out the electron beam. Several research groups have successfully adopted this approach to modify their linacs to produce FLASH dose‐rate electron beams.^4^, ^8^ Additionally, IntraOp has converted their mobile intraoperative unit (Mobetron, IntraOp, Sunnyvale, CA) for electron FLASH (eFLASH).^3^ Prior to our study, there was no commercially available FLASH solution that could serve as a widely accessible and cost‐effective modification for conventional linacs.

In our study, a commercial linear accelerator (Clinac 23EX, Varian Medical Systems, Palo Alto, CA) was converted to include an electron FLASH beam by Varian, a Siemens Healthineers company (Palo Alto, CA). The converted system (the “Clinac‐FLEX system”) provides the capability to deliver a broad 16 MeV electron beam at either FLASH or conventional dose rates for research projects. Varian's Clinac‐FLEX system has the potential to greatly increase access to ultra‐high‐dose‐rate delivery systems for FLASH radiation researchers and promote further multi‐institutional collaborations. Our institution, Faith Regional Carson Cancer Center in Norfolk, Nebraska, and Varian collaborated on the first installation of the Clinac‐FLEX system. The purpose of this study was to conduct the initial acceptance, commissioning, and dosimetric characterization of the Clinac‐FLEX system for cell and animal research using primarily radiochromic film, optically stimulated luminescent dosimeters, and plane‐parallel ionization chamber.

## 2 MATERIAL AND METHODS

### 2.1 Clinac‐FLEX system

Our installation of Varian's novel Clinac‐FLEX solution comprises a Clinac 23EX accelerator previously used clinically for photon (6 and 15 MV) and electron (6, 9, 12, 16, and 20 MeV) external beam radiotherapy, modified for eFLASH delivery. After installing the FLEX system, the modified Clinac 23EX is now capable of delivering a 16 MeV beam with FLASH dose rates while still maintaining the ability to deliver four of the conventional electron energies (i.e., 6, 9, 12, and 16 MeV). The conventional 20 MeV electron energy was replaced with the 16 MeV electron FLASH mode.

**FIGURE 1:**
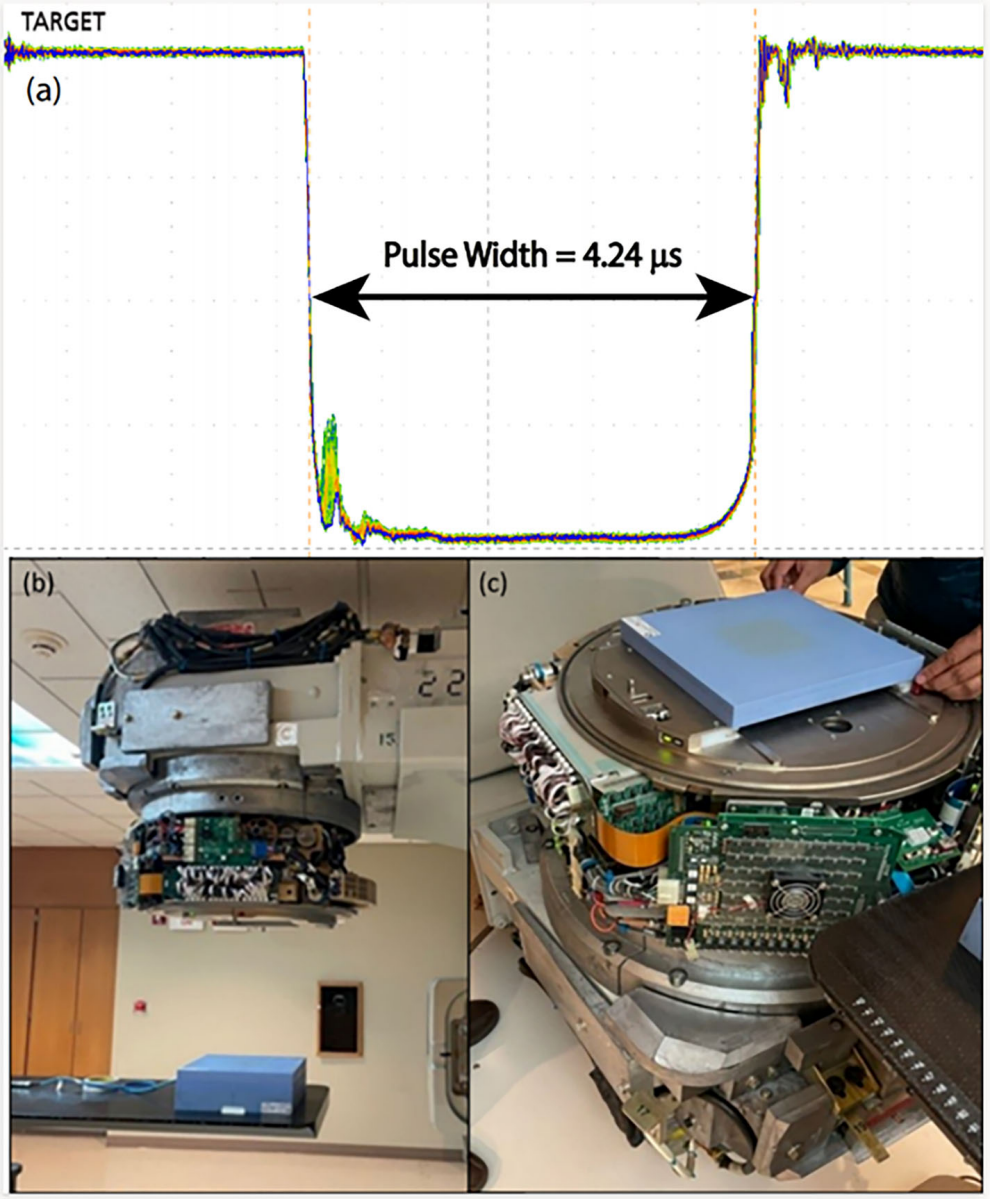
Displayed in the top image (a) is a representative FLASH beam pulse train, consisting of 99 successive pulses, measured by monitoring the current generated at the x‐ray target using an oscilloscope (MSO54, Tektronix) synchronized with Linac pulses (Note that the x‐ray target, which stays retracted for electron modes, was inserted during this measurement for beam tuning and diagnosis purposes). Within this pulse train, the mean pulse width was determined to be 4.242 ± 0.002 μs, while the pulse area, serving as a surrogate for dose‐per‐pulse, exhibited a variation of 1.3% [(max‐min)/mean]. Displayed in the bottom images are the experimental setups for the acceptance tests at gantry angles of (b) 0 degrees and (c) 180 degrees.

Varian's team made several substantial modifications to the linear accelerator to generate a 16 MeV electron beam with ultra‐high‐dose‐rates. First, after removing the x‐ray target, the RF power and electron gun systems were adjusted; specifically, the incoming RF power was changed to address the increased beam loading from the high‐current electron beam. Second, a thin scattering foil designed for a low electron energy was used to minimize the loss in dose rate while maintaining a relatively flat beam profile. Third, the Beam Pulse Counter was installed for FLASH delivery mode and enabled the user to deliver a discrete number of pulses (1 to 99 pulses per delivery) with a pulse width of 4.2 μs (Figure 1a). Additionally, the user can select a repetition rate (pulses per second) from seven discrete values (18 pulses/s to 180 pulses/s). This is unique in that the dose output is set by the number of pulses rather than the monitor units measured by the ion chamber inside the linac head because of the known saturation issue of the ion chamber at ultra‐high‐dose‐rates. Therefore, there is no active dose control available through the monitor unit chamber for eFLASH. The mode of operation for the conventional dose rate (CONV) electron beams remains available, and the linear accelerator can switch between FLASH and CONV modes of operation. After these modifications, the linear accelerator may only be used for research purposes due to vendor restrictions and the lack of active dose control for eFLASH.^13^

Varian's user guide for the FLEX system^14^ outlines the process for transitioning to the FLASH delivery mode. First, FLASH is delivered only in service mode, and therefore, additional quality assurance tests should be performed (e.g., thorough checklist, quality assurance of dose rate and beam constancy) applying the guiding principles from relevant AAPM Task Groups^15^, ^16^, ^17^ until a FLASH‐specific task group report is published. When transitioning to FLASH mode, specific settings need to be configured for the FLASH beam delivery. For example, all steering servos are deactivated, and approximately ten other interlocks are overridden to prevent any interference with the FLASH radiation delivery. Furthermore, the FLASH beam is its own separate electron energy configuration, and therefore, we sacrificed the 20 MeV electron mode for the 16 MeV electron FLASH mode. This retains the use of the conventional dose rate electron energies (6, 9, 12, 16 MeV), each having separate energy configurations.

The design specifications for the 16 MeV eFLASH beam were similar to those of the 16 MeV CONV beam, with a few distinct features. The 16 MeV CONV and FLASH beams were both configured to have an equivalent range at 50% of the maximum dose (*R_50_*) and a similar depth of maximum dose, *d_max_*, approximately at 3 cm. For the 16 MeV FLASH beam profile, the Varian protocol specifies that for a jaw‐defined 10 × 10 cm^2^ open field with the detector positioned at *d_max_* and 100 cm source‐to‐axis distance (SAD), the symmetry should be <3% and the off‐axis intensity (OAI) relative to that at the central axis should be >80% at ± 4.0 cm in both cross‐plane and in‐plane orthogonal directions. Additionally, the dose‐per‐pulse should be >1 Gy/pulse at the isocenter and the dose rate >500 Gy/s at the gantry upper accessory mount (source‐to‐surface distance (SSD) of ∼59 cm). The maximum instantaneous dose rate can be modified by changing the SSD. The time‐averaged dose rate for the delivery of multiple pulses can be altered by adjusting the SSD and/or selecting a different pulse repetition rate (18 pulses/s to 180 pulses/s). These and other eFLASH beam characteristics were tested during the acceptance testing and dosimetry characterization.

### 2.2 Installation, acceptance, and dosimetric characterization of the Clinac‐FLEX system

Immediately after the FLEX conversion of the linac to operate with 16 MeV eFLASH, a radiation survey was conducted to ensure safety. Next, acceptance tests and additional dosimetric characterization of the 16 MeV eFLASH beam were conducted based on the guiding principles for conventional radiotherapy.^18^, ^19^, ^20^

#### 2.2.1 Radiation survey

The radiation survey was conducted in the surrounding areas of the Clinac‐FLEX vault to assess the installed shielding for both photons and neutrons produced by the 16 MeV eFLASH beam. This was done to verify that the shielding design was sufficient for the purposes of radiation safety.^21^ The NCRP 151 report defined shielding criteria as 100 μSv/week for controlled areas and 20 μSv/week for uncontrolled areas.^21^ Furthermore, the Nuclear Regulatory Commission mandates that the radiation dose in an unrestricted area be less than 20 μSv in any hour.^22^

The Ludlum Model 9‐3 ion chamber and Ludlum 12‐4 neutron meter were used for measuring the photon and neutron components, respectively. Measurements for the dose rates were conducted at 91.4 cm above the floor and 30.5 cm away from outside the barrier or wall. For the areas above the vault, dose rates were measured at the floor surface and 91.4 cm above the floor. The delivered dose rate was approximately 180 Gy/s at the isocenter using the 180 Hz pulse repetition rate. The gantry angle was set to 0, 90, 180, and 270 degrees (in IEC scale and thereafter). To assess stray neutron radiation, the jaws were completely closed to generate the worst‐case scenario for neutron production. Similarly, to create the worst‐case scenario for secondary scattered radiation, a scattering material greater than 30 cm of tissue equivalent material (i.e., water‐filled plastic containers) was placed at the isocenter and irradiated using a field size of 40 × 40 cm^2^. No scattering material was used for assessing the primary radiation, and the field size was 40 × 40 cm^2^. The beam‐on time used for obtaining the dose rates was 10 s with the Beam Pulse Counter overridden. The occupied area for the Varian personnel was defined as the console area, and the occupancy factors (Table 2) defined by NCRP Report No. 151 were applied to other areas surrounding the vault.^21^

#### 2.2.2 Acceptance tests

Several acceptance tests were performed by Varian medical physicists and engineers, as well as by UNMC medical physicists. First, the dose at the isocenter was measured by positioning a plane‐parallel ion chamber (Advance Markus Electron Chamber, PTW, Freiburg, Germany) at 100 cm SAD in water‐equivalent plastic (Solid Water HE, Sun Nuclear Corporation, Melbourne, FL, USA) at 3‐cm depth with 10‐cm for backscatter (the continuous slowing down approximation range for 16 MeV electrons is around 7.63 cm). The field size was 10 × 10 cm^2^ without any cones or applicators, and the gantry angle was 0 degrees. Additionally, the OAI and symmetry of the beam profile were evaluated by translating the detector using the motorized couch by ± 4 cm in both the in‐plane and cross‐plane directions, which correlates to 80% of the field width for a conventional electron beam.^15^ Flatness and symmetry were also evaluated in a subsequent setup using various electron applicators and radiochromic film.

Lastly, the gantry was rotated to 180 degrees, and the water‐equivalent plastic was placed directly on the head of the Clinac‐FLEX machine to measure the maximum dose rate. The plane‐parallel chamber was inverted such that the entrance window of the detector faced the incoming electron beam. The chamber was positioned at 3 cm depth in water‐equivalent plastic with the field size set to 10 × 10 cm^2^ (no applicator). These two setups (gantry 0 and 180 degrees) are shown in Figure 1b,c.

#### 2.2.3 Dosimetric characterization of Clinac‐FLEX system

The dosimeters used in this study included a plane‐parallel ionization chamber (Advance Markus Chamber Type 34045, PTW, Freiburg, Germany), radiochromic film (GafChromic EBT‐XD, Ashland Inc., Wayne, NJ, USA), and optically stimulated luminescent dosimeters (OSLDs) (NanoDot, Landauer Inc., Glenwood, Il). The vented plane‐parallel ionization chamber has approximately a 1 mm gap between the parallel plates, an active volume of 0.02 cm^3^, high spatial resolution, and a useful voltage range of ± 50 to 300 V (maximum of ± 400 V). Regarding the specifications of the chamber's ion collection efficiency at the nominal voltage (i.e., ± 300 V), the maximum dose‐per‐pulse is 2.78 and 5.56 mGy for ≥99.5% and ≥99.0% saturation, respectively.^23^

The radiochromic film used in this study is designed to measure the high absorbed dose with a high spatial resolution (i.e., 0.35 mm).^24^ It has a dynamic dose range of 0.1 to 60 Gy with an optimal dose range of 0.4 to 40 Gy using the triple‐channel calibration protocol. The energy dependency of the radiochromic film is less than 5% when exposed between 100 keV and 18 MeV.^25^ Additionally, radiochromic film has been shown to be dose‐rate independent up to 2 × 10^4^ Gy/s.^26^, ^27^ The film was digitized with red‐green‐blue channels using a transmission‐mode flatbed scanner (Epson Expression 12000 XL photo scanner, Suwa, Nagano, Japan) with a glass compression plate and analyzed with commercial software (FilmQApro, Version 7, Ashland Inc., Wayne, NJ, USA). The triple‐channel calibration curve was generated using eleven films that were irradiated to known doses (i.e., 0, 63, 125, 250, 500, 750, 1000,1250, 1500, 2000, and 4000 cGy) using a 15 MeV CONV electron beam emitted by a clinical linear accelerator (TrueBeam, Varian Medical Systems, Palo Alto, CA).

The OSLDs used in this study were made of aluminum oxide powder doped with carbon (Al_2_O_3_:C). OSLDs exhibit an energy dependence of 5% in the range of 5−20 MeV for photons and electrons.^28^, ^29^, ^30^ Per the vendor's instructions, the manufactured OSLDs have a linear response with an absorbed dose of up to 3 Gy and a non‐linear response of up to 15 Gy. Therefore, a linear calibration curve was applied between 0.15 to 3 Gy, and a non‐linear calibration curve was applied between 3 to 15 Gy.^31^ Additionally, OSLDs have a simple read‐out process, can reach stable signal output after 8 min post‐irradiation, and have low signal loss.^32^, ^33^ Furthermore, OSLDs are largely dose‐rate independent (<2%) up to 4 × 10^9^ Gy/s.^34^

Summarized in Table 1 are the various dosimetry tests that were performed to characterize the 16 MeV eFLASH beam for radiobiology studies. First, the plane‐parallel chamber was used to characterize the pulse linearity. The chamber was positioned in water‐equivalent plastic at 3 cm depth with 10 cm of backscatter at 59.5 cm SSD, gantry at 0 degrees, and field size of 10 × 10 cm^2^ (no applicator). The pulse repetition rate was set to the maximum repetition rate, that is, 180 pulses/s, and the number of delivered pulses was varied from 1 to 99 pulses. Second, the relationship between the dose and distance from the radiation source was characterized. The absorbed dose was measured with radiochromic film and OSLDs, and the charge was measured by a plane‐parallel chamber. These dosimeters were positioned at 3 cm depth in water‐equivalent plastic with 10 cm for backscatter, and the SSD was varied from 59.5 to 129 cm. Similar to the prior pulse linearity study, the setup was a field size of 10 × 10 cm^2^, gantry at zero degrees, repetition rate of 180 pulses/s, and delivery of 10 pulses. Third, the change in output with respect to the field size was measured with the plane‐parallel chamber for field sizes of 5 × 5 cm^2^ to 40 × 40 cm^2^ without an applicator. The chamber was positioned in water‐equivalent plastic at 3 cm depth with 10 cm of backscatter, 100 cm SSD, gantry at zero degrees, a repetition rate of 180 pulses/s, and a single pulse was delivered for each measurement.

**TABLE 1:** Summary of dosimetric tests for characterization of the 16 MeV eFLASH beam for radiobiology studies.

Test	Content
Pulse linearity	Measured 1 to 99 pulses with plane‐parallel chamber
Inverse square law	Measured absorbed dose with radiochromic film and OSLDs Measured charge with plane‐parallel chamber Variable source‐to‐dosimeter distance from 62.8 to 132 cm with virtual source correction
Field size effect	Measured the relative change in output with field size Field sizes of 5 × 5 cm^2^ to 40 × 40 cm^2^ without an applicator
PDDs and profiles	Measured PDDs and profiles with radiochromic film Fields sizes included 6 × 6 cm^2^ applicator with 2 cm diameter circular cutout; 6 × 6 cm^2^ applicator; 15 × 15 cm^2^ applicator; and 40 × 40 cm^2^ field size without applicator

To measure percent depth dose (PDD) curves and profiles of the 16 MeV CONV and eFLASH electron beams, radiochromic film was placed in water‐equivalent plastic and positioned parallel and perpendicular to the central axis (CAX) of the beam, respectively. For measuring PDDs in water‐equivalent plastic, there was a need to minimize the impact of the air gap on CAX. For this reason, we placed a bolus between the film and the water‐equivalent plastic blocks and applied C‐clamps to minimize this air gap. Furthermore, a slight rotation in the gantry was applied (3 degrees) to further minimize the effect of the air gap.^35^ This procedure was not necessary for profiles for which the film was placed perpendicular to the beam CAX at 3 cm depth in the water‐equivalent plastic with 10 cm of backscatter. Additional profiles were measured at 1, 3, and 6 cm depth for the 10 × 10 cm^2^ applicator. For the measurements with eFLASH, the setup was a field size of 10 × 10 cm^2^ (with and without an applicator), SSD of 100 cm, a repetition rate of 180 pulses/s, and delivery of 40 pulses. For the CONV electron beam, 540 MUs were delivered at a dose rate of 600 MU/min using the identical field size, SSD, and depth as the measurements with eFLASH. Additional PDDs and off‐axis ratios (OARs) were investigated for various field sizes (6 × 6 cm^2^ applicator; 6 × 6 cm^2^ applicator with 2 cm diameter circular cutout; 15 × 15 cm^2^ applicator; and 40 × 40 cm^2^ field size without the applicator) for both the 16 MeV eFLASH and CONV electron beams. For eFLASH and CONV electron beams, the R_50_ values were determined from the PDDs of the 10 × 10 cm^2^ field size (with and without an applicator). Moreover, penumbra and lateral dose‐rate gradient values were calculated for both eFLASH and CONV electron beams at 3‐cm depth for various applicator sizes, including 15 × 15 cm^2^, 10 × 10 cm^2^, 6 × 6 cm^2^, and 6 × 6 cm^2^ applicator with a 2 cm diameter circular cutout. The lateral dose‐rate gradient (% per mm) was calculated as the average dose‐rate falloff per distance between 80% and 20% of the maximum dose rate.

#### 2.2.4 Stability test

The beam stability of the Clinac‐FLEX system was evaluated once per week for approximately a 4‐month period immediately following the commissioning of the machine. For the setup of this experiment, the dosimeter was positioned at 3 cm depth in water‐equivalent plastic with 10 cm for backscatter, SSD of 100 cm, gantry at zero degrees, and field size of 10 × 10 cm^2^ with no applicator. The repetition rate and number of pulses varied from 18 to 180 pulses/s and 1 to 99 pulses, respectively. Both the plane‐parallel chamber and radiochromic film were used for these tests to isolate the source of instability. The percentage deviations from the daily average output were determined using both dosimeters. Approximately 1‐year after the FLEX conversion, Varian added a new procedure for FLASH delivery. This involved turning off the pulse forming network (PFN) servo, which is turned on for CONV delivery. The PFN servo is responsible for controlling the high‐voltage electrical pulses that power the accelerator's electron beam generation system and enable the rapid discharge of stored energy from the PFN into the accelerator's waveguide structure. In the linac console cabinet, there is a physical toggle switch for the PFN servo. If the switch is directed toward the left side, it is in the “off” position for the FLASH mode, and if it is directed toward the right side, it is in the “on” position for the CONV mode. The output stability was re‐evaluated after switching off the PFN servo for FLASH delivery. Varian specifies that the stability of the delivered dose should be greater than 95% between pulses.^14^ Additionally, the effect of repetition rate on the beam stability was evaluated as well.

#### 2.2.5 Plane‐parallel ionization chamber polarizing voltage effect

To characterize the polarity effects of the plane‐parallel chamber in the eFLASH environment, we measured the charge at different polarizing voltages (i.e., ± 100, ± 150, ± 300, ± 400, ± 450 V). The dosimeter was positioned at 3 cm depth in water‐equivalent plastic with 10 cm of backscatter, SSD of 100 cm, gantry at zero degrees, repetition rate of 180 pulses/s, and delivery of one pulse.

#### 2.2.6 Plane‐parallel ionization chamber recombination study

Ionization chambers are known to exhibit substantial recombination effects when used in high‐dose‐rate conditions, resulting in lower ion collection efficiency.^36^ As per the manufacturer, the advanced Markus chamber can collect ions with acceptable efficiency in conventional high‐dose‐rate beams.^23^ However, the advanced Markus chamber has a notable loss of efficiency when used in ultra‐high‐dose‐rate beams, warranting the need for characterization in the FLEX environment.^37^, ^38^

To characterize the ion recombination effect of the plane‐parallel chamber under eFLASH conditions, we measured the dose‐per‐pulse and charge‐per‐pulse using both radiochromic film and plane‐parallel chamber at varying SSDs. The dosimeters were positioned at 3 cm depth in water‐equivalent plastic with 10 cm of backscatter, gantry at zero degrees, field size of 10 × 10 cm^2^ (no applicator), repetition rate of 180 pulses/s, and delivery of 50 pulses. The data was plotted as ion collection efficiency versus dose‐per‐pulse. The ion collection efficiency was estimated by comparing the dose‐per‐pulse of the plane‐parallel chamber in relation to the reference dose‐per‐pulse determined by the radiochromic film. Next, various models were applied to resolve the ion recombination effect. In addition to the classical model for the plane‐parallel ionization chamber formulated by Boag,^39^ an extended model^40^ including the free electron component as well as the logistic model^37^ were applied.

### 2.3 Cell lines, cell treatment, and cell analysis

By way of introduction, we studied the effect of the FLASH dose rate (180 Gy/s) and non‐FLASH dose rate (18 Gy/s) on breast normal and cancer cells. To generate the two different dose rates for the cell experiment, we operated the Clinac‐FLEX system's 16 MeV electron FLASH beam at the highest and lowest repetition rates (i.e., 180 pulses/s and 18 pulses/s, respectively). A custom 3D‐printed cell holder was designed to reduce the setup uncertainty and air gaps.^41^ The 3D‐printed cell holder was positioned such that the cells were at 3 cm depth with 10 cm of backscatter using water‐equivalent plastic and bolus to reduce air gaps. Additionally, the setup for this experiment was gantry at zero degrees, SSD of 100 cm, field size of 10 × 10 cm^2^ (no applicator), and the number of pulses varied such that 0 Gy (control) or 5 Gy was delivered to the cells. The absorbed dose at the location of the cells was confirmed with radiochromic film prior to delivery, and in vivo film dosimetry was conducted during each cell irradiation as a means of quality assurance.

#### 2.3.1 Cell lines and cell culture

Human breast cancer cell line BT‐549 was obtained from the American Type Culture Collection (Manassas, VA) and maintained in Dulbecco's Modified Eagle's Medium (DMEM) containing 10% fetal bovine serum (FBS). 76N is a line of primary human mammary normal epithelial cells immortalized by human telomerase (hTERT).^42^ 76N cells were maintained in Mammary Epithelial Growth Medium (MEBM) supplemented with Bullet Kit from Lonza Bioscience (Morrisville, NC) and 1% FBS.

#### 2.3.2 Cell treatment and analysis

For radiation treatment, exponentially growing cells were seeded and incubated for 24 h before radiation exposure. The cells were irradiated under normoxic conditions. The irradiated cells were subsequently incubated for 4 days and analyzed for cell morphology with an IMT‐2 Olympus phase contrast microscope (Tokyo, Japan). For the viability study, the irradiated cells were incubated for 14 days and analyzed for cell survival by cell viability assay, as described previously.^43^ In brief, the surviving cells were fixed in Methanol for 10 min, visualized by staining with crystal violet (Sigma‐Aldrich, St. Louis, MO), scanned using an EPSON Perfection 4490PHOTO scanner, quantified by ImageJ (NIH) software,^44^ and analyzed with SigmaPlot software (SPSS Inc, Palo Alto, CA).

## 3 RESULTS

### 3.1 Radiation survey

For the personnel shielding criteria in the console area, no neutron dose rates exceeded NRC's instantaneous dose rate limit of 2.0 mrem/h.^22^ For photon dose rates in the console area, there were three gantry positions that exceeded 2.0 mrem/h, that is, 3.2 mrem/h, 2.4 mrem/h, and 6.0 mrem/h at gantry angles of 90 degrees, 180 degrees and 270 degrees, respectively (Table 2). However, these dose rates were determined with the Beam Pulse Counter overridden by the vendor to conduct the survey. In normal operating conditions, the maximum achievable dose rate in any given hour is much lower for the eFLASH beam. Specifically, there is a mandatory time gap (>10 s) between each eFLASH delivery due to the Beam Pulse Counter, and the maximum beam on‐time for eFLASH is short (assumed to be 0.5 s). Considering these two factors, the dose rate of 6 mrem/h is considerably reduced, and it would be exceedingly difficult to exceed the NRC limit of 2 mrem in any hour. Thus, this facility was determined to meet the personnel criteria for shielding the console area.

**TABLE 2:** Photon Survey results for facility shielding criteria based on NCRP Report No. 151. The controlled areas consist of the console area and the block room, whereas the uncontrolled areas include the hallway outside the room, the supply room, and the outside building.

Gantry angle	Location	Instantaneous dose rate	Occupancy factor	Dose rate using occupancy factor
0°	Console Area	1.2 mR/h	1	1.2 mrem/h
90°	Console Area Block Room Hallway outside Room	3.2 mR/h 150 mR/h 38 mR/h	1 1/5 1/5	3.2 mrem/h 30 mrem/h 8 mrem/h
180°	Console Area Supply Room (above)	2.4 mR/h 16 mR/h	1 1/20	2.4 mrem/h 0.8 mrem/h
270°	Console Area Outside Building (west)	6 mR/h 50 mR/h	1 1/40	6 mrem/h 1.25 mrem/h

Table 2 summarizes the pertinent results of the photon survey considering different gantry angles and various locations. At a gantry angle of 0 degrees, no measured instantaneous dose rate exceeded 2 mrem/h for any location outside of the vault. For gantry angles other than 0 degrees, the worst‐case scenario was the block room (controlled area) with the eFLASH beam directed at it (gantry rotated to 90 degrees). This resulted in a dose rate of 30 mrem/h after considering the 1/5 Occupancy Factor. Assuming that the radiobiology experiments are only conducted 1 day per week, an 8‐h workday, and the operational eFLASH time is 0.5 s with a mandatory time gap (>10 s) between deliveries, both the weekly dose rate and the dose rate in 1 h were considerably reduced resulting in <10 mrem/week and <2 mrem/h, respectively. Furthermore, the radiobiology experiments are done at a gantry angle of 0 degrees (as this is the specific gantry angle that Varian guarantees output and symmetry of the eFLASH beam); therefore, the Use Factor for adjacent primary barriers could have also been reduced for these calculations as the FLEX system was located on the ground floor with no basement. Thus, the shielding criteria defined by NCRP Report No.151 were conservatively met, and area radiation badges will be positioned at various locations surrounding the vault for radiation monitoring.

### 3.2 Acceptance tests

During acceptance testing of the Clinac‐FLEX system, the measured dose‐per‐pulse at the isocenter was 1.270 Gy/pulse (Figure 1b). The maximum measured dose‐per‐pulse was 3.8 Gy/pulse (i.e., dose rate of 689.6 Gy/s) for which the gantry was at 180 degrees and the detector was positioned near the accessory mount level, as illustrated in Figure 1c. Varian physicists attained these dose rate measurements using a charge‐to‐dose response curve obtained for their plane‐parallel chamber (Advance Markus Chamber Type 34045, PTW, Freiburg, Germany) corrected for ion recombination^45^ which was confirmed using OSLD and radiochromic film (GafChromic EBT3, Ashland Inc., Wayne, NJ, USA).

Flatness and symmetry of the eFLASH profile were evaluated using a series of point dose measurements with the plane‐parallel chamber at *d_max_* for the 10 × 10 cm^2^ field size (no applicator). The average OAI was 86.1 ± 0.8% at a distance of ± 4.0 cm orthogonal to CAX, and symmetry was within ± 1.1%. Both agreed with Varian's specifications and the American Association of Physicists in Medicine's (AAPM's) Task Group 40 (TG‐40) recommendations.^46^ In subsequent experiments, flatness and symmetry measurements were conducted with radiochromic film for additional field sizes useful for radiobiology studies.

### 3.3 Dosimetric characterization of Clinac‐FLEX system

**FIGURE 2:**
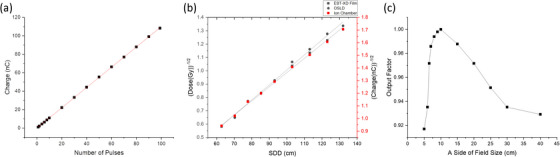
Dosimetric tests for (a) pulse linearity, (b) inverse square law, and (c) field size effect.

Displayed in Figure 2 are three eFLASH dosimetry tests that were performed with the Clinac‐FLEX system. First, the collected charge was linear (or proportional) to the number of pulses delivered with *R* ^2^= 0.9996 (Figure 2a), confirming the pulse linearity of the FLEX system. Second, after correcting for the virtual‐source distance, the 16 MeV eFLASH beam followed the inverse square law as measured by radiochromic film and OSLDs with *R* ^2^ = 0.9972 and *R* ^2^ = 0.9955, respectively (Figure 2b). Additionally, the measurements with radiochromic film and OSLDS agreed dosimetrically within 3%. Third, the relative output factor increased with increasing field size from 5 × 5 cm^2^ to 10 × 10 cm^2^ and then decreased with increasing field size from 10 × 10 cm^2^ to 40 × 40 cm^2^ (Figure 2c) due to the change in radiation scattering properties introduced by the jaws and the irradiated volume of water‐equivalent plastic.^47^

**FIGURE 3:**
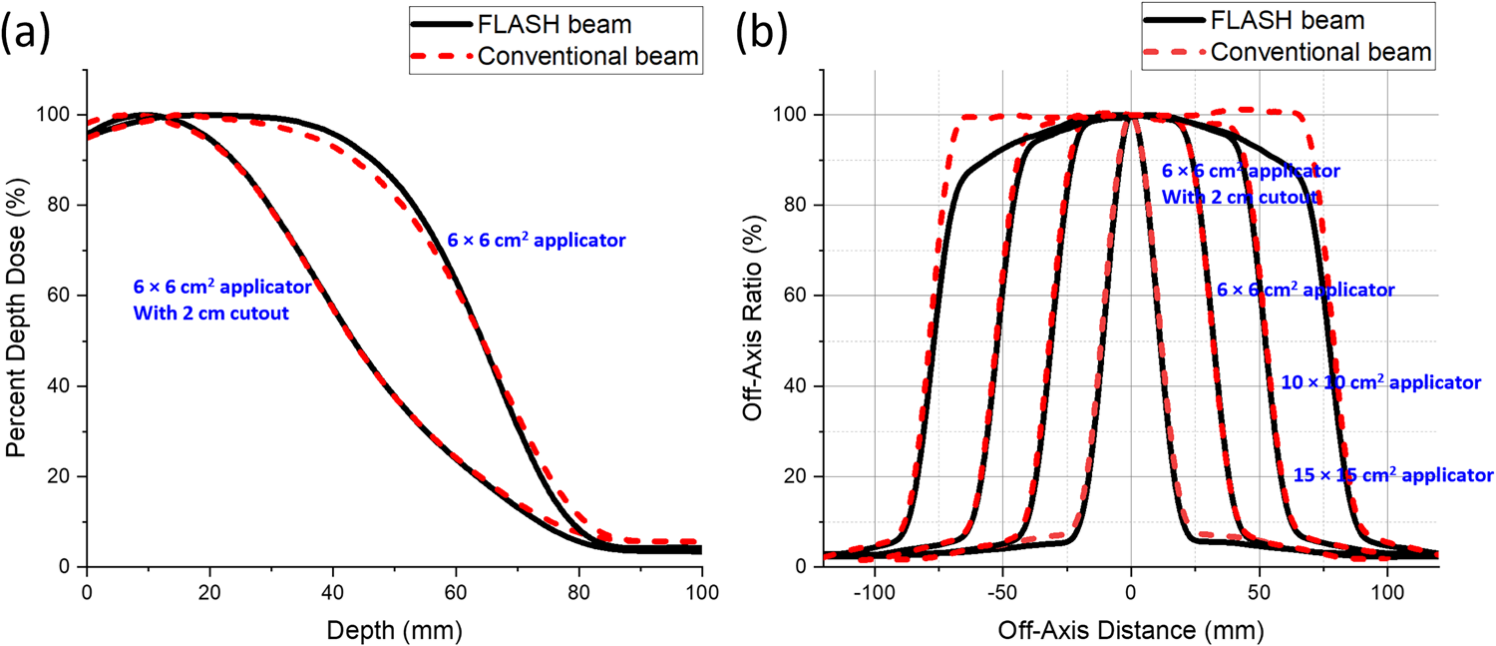
Depicts 16 MeV FLASH and conventional electron (a) percent depth dose curves for 6 × 6 cm^2^ applicator with 2 cm diameter circular cutout (left) and 6 × 6 cm^2^ applicator (right), and (b) relative cross‐plane profiles at 3‐cm depth for 6 × 6 cm^2^ applicator with 2 cm diameter circular cutout, 6 × 6 cm^2^ applicator, 10 × 10 cm^2^ applicator, and 15 × 15 cm^2^ applicator.

**FIGURE 4:**
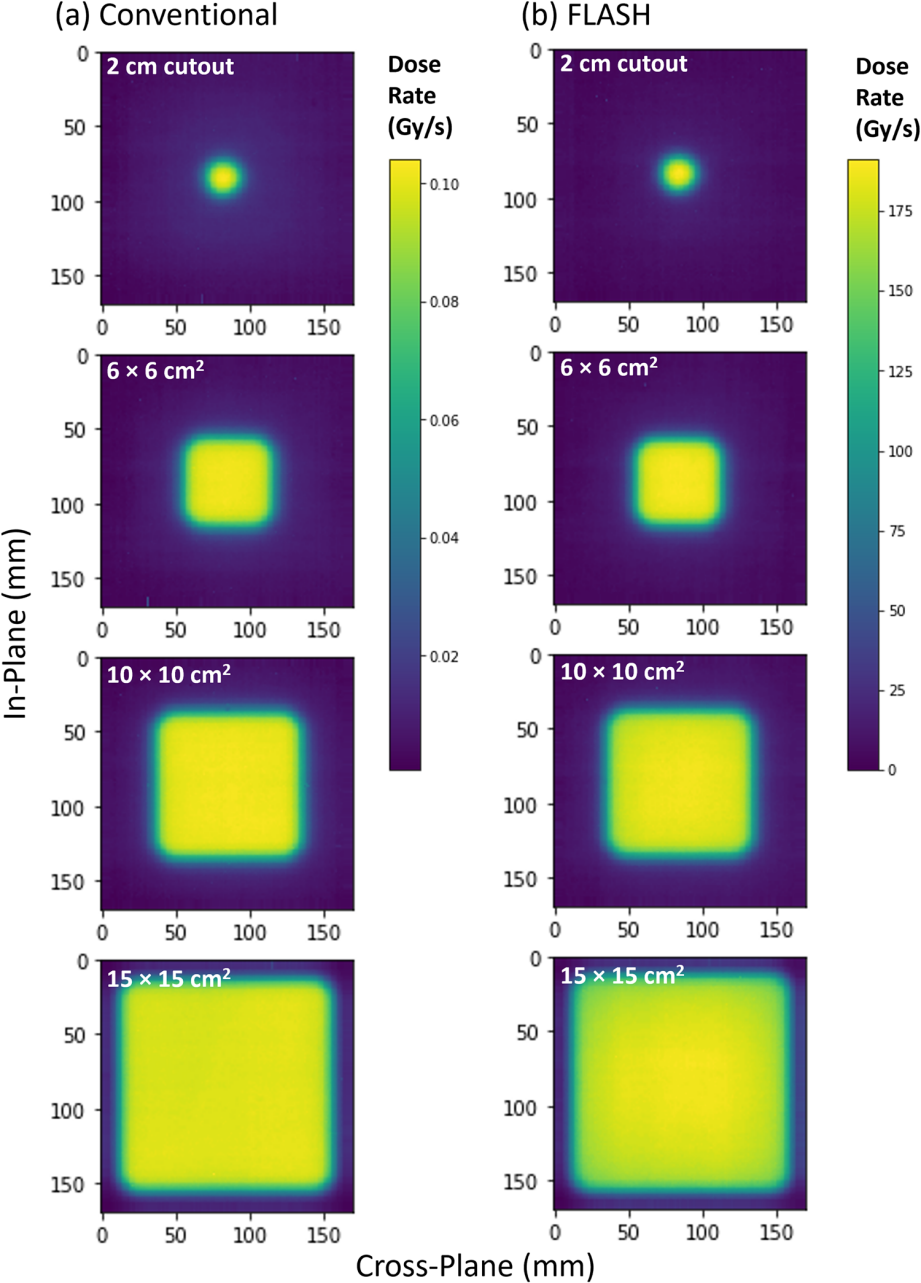
Two‐dimensional (2‐D) dose‐rate distributions for (a) conventional and (b) FLASH electron beams at 3‐cm depth for 6 × 6 cm^2^ applicator with 2 cm diameter circular cutout, 6 × 6 cm^2^ applicator, 10 × 10 cm^2^ applicator, and 15 × 15 cm^2^ applicator. For example, the measured dose rate with the 10 × 10 cm^2^ applicator at 100 cm SSD, 3 cm depth, on the central axis (CAX) for the conventional electron beam was 0.1 Gy/s, whereas the dose rate significantly increased to 190 Gy/s when utilizing the eFLASH beam.

The radiochromic film measurements of PDDs and profiles for the 16 MeV eFLASH and CONV electron beams were consistent, as demonstrated in Figure 3, Figure 4, and Table 3. The average and maximum (MAX) percent differences between the PDDs of the eFLASH and CONV mode for the 6 × 6 cm^2^ applicator with 2 cm diameter circular cutout, 6 × 6 cm^2^ applicator, 10 × 10 cm^2^ applicator, 15 × 15 cm^2^ applicator, and 40 × 40 cm^2^ open field (no applicator) were −0.61 ± 0.81% (MAX: 2.33% at 0.07 cm depth), 0.43 ± 2.12% (MAX: 3.43% at 7.62 cm depth), −0.10 ± 1.26% (MAX: 2.39% at 7.69 cm depth), 0.37 ± 1.08% (MAX: 1.54% at 8.40 cm depth), and −0.29 ± 1.43% (MAX: 2.81% at 0.99 cm depth) on average, respectively. As the field size increased, the surface dose decreased while *d_max_* and *d_90_* (i.e., depth at 90% of the maximum dose) increased and shifted to deeper depths. The *R_50_* values determined from the PDDs of the 10 × 10 cm^2^ applicator and 10 × 10 cm^2^ open field (no applicator) agreed within 0.05 cm between the 16 MeV eFLASH and CONV electron beams (Table 3). Furthermore, the flatness for the FLASH and CONV profiles agreed within 0.5% for field sizes less than 10 × 10 cm^2^ (Figure 3b). However, for the larger applicators, the flatness began to diverge (e.g., 15 × 15 cm^2^), as displayed in Figure 3b. Specifically, the difference in flatness for FLASH and CONV beams of the 10 × 10 cm^2^ and 15 × 15 cm^2^ field sizes were 1.5% and 6.3%, respectively. In general, the profiles for both FLASH and conventional dose rates agreed; however, the FLASH beam profile was less flat compared to the conventional beam profile as the field size increased >10 × 10 cm^2^ (Figure 3b). Moreover, the two‐dimensional (2‐D) dose‐rate distributions demonstrated a similar trend for both eFLASH and CONV electron beams at 3‐cm depth for the 6 × 6 cm^2^ applicator with 2 cm diameter circular cutout, 6 × 6 cm^2^ applicator, 10 × 10 cm^2^ applicator, and 15 × 15 cm^2^ applicator (Figure 4).

**TABLE 3:** *R_50_* determined from the PDDs of the 10 × 10 cm^2^ applicator and 10 × 10 cm^2^ open field (no applicator) for 16 MeV eFLASH and CONV electron beams.

	*R_50_* (cm)	
Dose rate	Applicator	No applicator
Conventional	6.35	6.35
FLASH	6.37	6.40

Table 4 compares the penumbra and lateral dose‐rate gradient values for both the conventional and FLASH electron beams at 3 cm depth for the following field sizes: 6 × 6 cm^2^ applicator with a 2 cm diameter circular cutout, 6 × 6 cm^2^ applicator, 10 × 10 cm^2^ applicator, and 15 × 15 cm^2^ applicator. For the conventional beam, both the penumbra and lateral dose‐rate gradient values remained similar as the applicator size increased. However, for the eFLASH beam, the penumbra increased, and the lateral dose‐rate gradient decreased as the applicator size increased.

**TABLE 4:** Penumbra (mm) and lateral dose‐rate gradient (% per mm) comparison for conventional and FLASH electron beams at 3‐cm depth for 6 × 6 cm^2^ applicator with 2 cm diameter circular cutout, 6 × 6 cm^2^ applicator, 10 × 10 cm^2^ applicator, and 15 × 15 cm^2^ applicator.

	Penumbra (mm)		Lateral dose‐rate gradient (%/mm)	
Applicator size	Conventional	FLASH	Conventional	FLASH
2 cm cutout	10.7	10.2	5.6	5.9
6 × 6 cm^2^	11.3	11.4	5.3	5.3
10 × 10 cm^2^	11.4	12.2	5.3	4.9
15 × 15 cm^2^	11.2	14.0	5.4	4.3

### 3.4 Stability test

**FIGURE 5:**
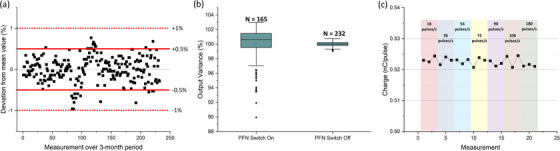
Beam stability output measurements for (a) the 3‐month observation period after turning off the PFN servo for FLASH delivery, (b) PFN servo “On” versus “Off,” and (c) various repetition rates (18–180 pulses/s).

Initially, to assess the stability of the system, 165 output measurements were acquired with the plane‐parallel chamber over the 4‐month period immediately following the commissioning of the Clinac‐FLEX system. A total of seven and eleven measurements deviated by more than 5% and 3% from the mean, respectively. The resulting standard deviation was 2.48% over the 4‐month period. A similar variance in the beam output was observed with radiochromic film. With support from Varian experts, we conducted a rigorous investigation of the initial output instability of the FLEX system. The output fluctuations were mitigated by turning off the PFN servo and providing a sufficient time gap between consecutive FLASH experiments (i.e., 100 seconds) which significantly improved the system performance (see Figure 5a,b).

After applying the updated procedure of turning off the PFN servo for FLASH delivery, the output stability significantly improved. With the PFN servo switch off, all 232 output measurements were less than ± 1% from baseline, and the standard deviation was 0.31% over the 3‐month period (Figure 5a). As shown in Figure 5b, the beam output deviation was within ± 1.0% with the PFN servo off. This was a remarkable improvement considering that the beam output occasionally would drop by up to 10% with the PFN servo on (Figure 5b).

Due to the FLASH effect occurring at ultra‐high‐dose‐rates exceeding 40 Gy/s, the repetition rate is a key factor to be considered. The output stability measured with the plane‐parallel chamber for various repetition rates is displayed in Figure 5c. The measurements demonstrated that the beam output was stable regardless of the repetition rate, with a mean charge of 0.52 nC/pulse and a relative standard deviation of 0.24% across all repetition rates. For the delivery of 10 pulses with the repetition rate of 18, 36, 54, 72, 90, 108, and 180 pulses/s, the collected charge was 5.233 ± 0.010, 5.229 ± 0.013, 5.227 ± 0.008, 5.225 ± 0.016, 5.220 ± 0.008, 5.232 ± 0.021, and 5.213 ± 0.003 nC/pulse, respectively.

### 3.5 Ionization chamber recombination study

**FIGURE 6:**
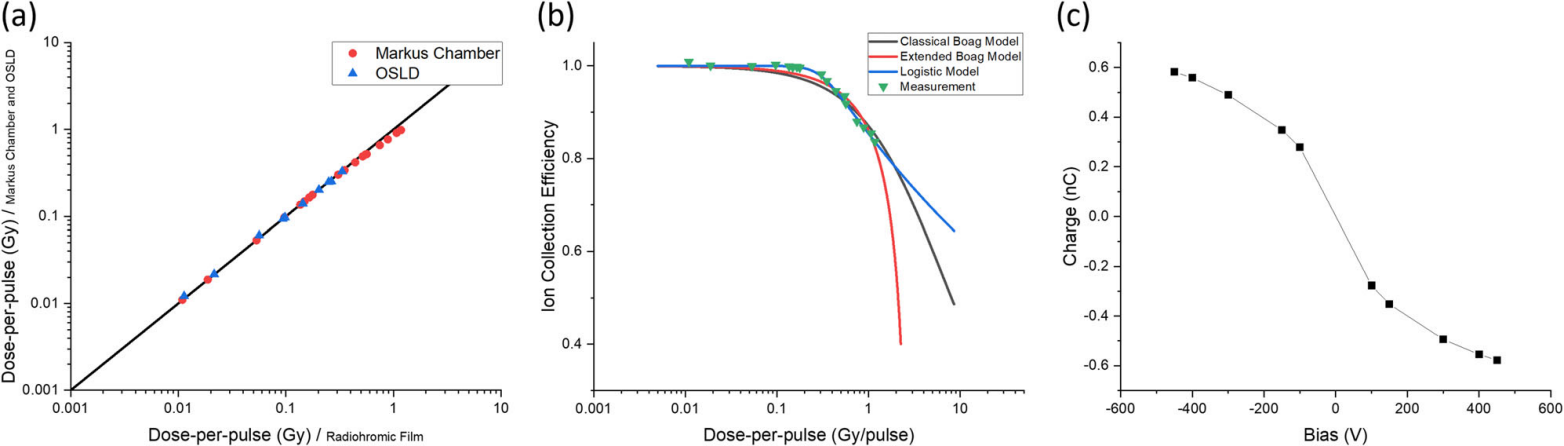
Displayed is (a) dose‐per‐pulse measured by plane‐parallel chamber and OSLDs versus dose‐per‐pulse measured by radiochromic film; (b) ion collection efficiency for the plane‐parallel chamber as a function of dose‐per‐pulse with the Boag's classical^39^ and extended models^40^ as well as the logistic model fitted to the measured data;^37^ and (c) collected charge of the plane‐parallel chamber as a function of bias voltage.

In Figure 6a, the absorbed dose measurements using both OSLDs and radiochromic film were in agreement, for which the mean absolute dose deviation was 0.48%, and the linear model fit the data well, *R* ^2^ = 0.999. For the plane‐parallel chamber, the dose‐per‐pulse curve was linear up to approximately 0.1 Gy/pulse but became saturated thereafter. Figure 6b shows the ion collection efficiency curve for the plane‐parallel chamber in the eFLASH environment with the corresponding Boag's classical and extended models, and the logistic model. The *R* ^2^ values for Boag's classical and extended models were 0.922 and 0.963, respectively, whereas the *R* ^2^ value for the logistic model was 0.993. Boag's model accuracy was improved with lower bias voltage, that is, 50 V, but agreement with measured data degraded with higher bias voltages, that is, 300 V. In general, the logistic function offered increased accuracy compared to the other models and is the widely accepted model for the ion collection efficiency for the plane‐parallel chamber.^37^

As demonstrated in Figure 6c, the collected charge of the plane‐parallel chamber increased exponentially with increasing bias voltage. It was observed that the saturation curve first rose linearly at low voltages and then saturated at higher voltages. This is because electric fields between electrodes become stronger as bias voltage increases, which enables the ion chamber to collect more charge until a plateau is reached.^48^ The polarization effect was characterized by conducting measurements with both polarities. The change in polarity resulted in less than a 1% change in charge collection, on average.

### 3.6 Dose rate effect of radiation on cell morphology and survival of breast normal and cancer cells

**FIGURE 7:**
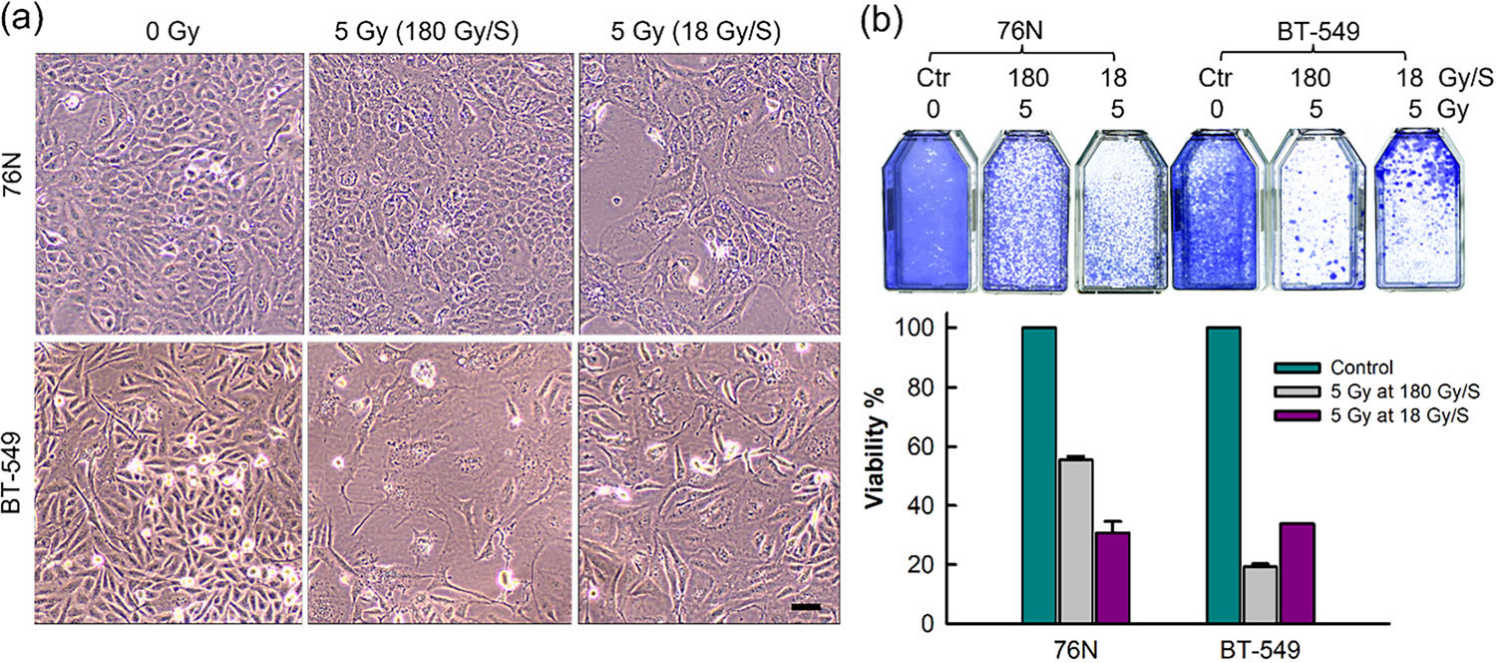
Breast normal (76N) and cancer (BT‐549) cells were exposed to 0 or 5 Gy radiation delivered either at 18 or 180 Gy/s dose rate. (a) The irradiated cells were incubated for 4 days and then imaged with an IMT‐2 Olympus phase‐contrast microscope. Scale bar = 1 μm. (b) The irradiated cells were incubated for 14 days, and the surviving cells were visualized by crystal violet staining, quantified by ImageJ software, and analyzed with SigmaPlot.

We compared the FLASH dose rate (180 Gy/s) with the non‐FLASH dose rate (18 Gy/s) for the effect on breast normal and cancer cells. As shown in Figure 7a, 5 Gy irradiation by 180 Gy/s showed negligible effect on the morphology of 76N breast normal cells as compared to the control unirradiated 76N cells (0 Gy), whereas 5 Gy irradiation by 18 Gy/s showed a noticeable effect on the morphology of 76N cells, with the cells becoming flattened and enlarged compared to the unirradiated control cells. Furthermore, FLASH‐irradiated 76N normal cells showed a better survival rate compared to 76N cells irradiated by the non‐FLASH dose rate (Figure 7b). We next examined the dose rate effect on BT‐549 triple‐negative breast cancer cells with the same method. As shown in Figure 7a, in contrast to 76N normal cells, FLASH‐irradiated BT‐549 breast cancer cells displayed flattened and enlarged morphology, while the non‐FLASH‐irradiated BT‐549 cells exhibited morphology more identical to the control unirradiated cells. The dose rate changes also affected the survival of BT‐549 cancer cells, which was also compared to 76N cells. The non‐FLASH irradiated BT‐549 cells showed similar cell survival to non‐FLASH‐irradiated normal cells; however, this observation needs to be confirmed with additional studies.

Moreover, FLASH‐irradiated 76N normal cells displayed a much higher survival rate than FLASH‐irradiated breast cancer cells (56% vs. 19%). Since the dose rate of radiation is the only variable examined in this experiment, these results suggest that the radiation dose rate plays an essential role in determining the fate of cellular response to radiation. In addition, FLASH irradiation showed better protection of normal breast cells than non‐FLASH radiation. Future studies are needed to identify the FLASH effect's biological mechanism and translational value.

## 4 DISCUSSION

Several research laboratories have modified linear accelerators to produce ultra‐high‐dose‐rates to study the FLASH effect (Table 5). However, these custom linacs are scarce and resource‐intensive. In our study, we performed the initial acceptance and commissioning of a novel research system that retrofitted a conventional linear accelerator with a 16 MeV eFLASH beam for cell and animal studies. Notably, this solution has the potential to be widely implemented and adopted such that more researchers can investigate the FLASH effect.

**TABLE 5:** Currently used electron FLASH system with their corresponding energies and reported dose‐rates.

Machine	Energy	Beam output	Location	Reference
Oriatron eRT6	6 MeV	∼200 Gy/s	100 cm SSD	Jaccard et al. (2017)^49^
Elekta Precise	8 MeV	∼1000 Gy/s	Wedge position (19 cm from target reference)	Lempart et al. (2019)^50^
Varian Clinac 21EX	20 MeV	∼900 Gy/s	Ion chamber position	Schuler et al. (2017)^51^
Varian Clinac 2100 C/D	10 MeV	310 Gy/s	isocenter	Rahman et al. (2021)^4^
Experimental LINAC	200 MeV	117 Gy/s	Not given	McManus et al. (2020)^52^
Research LINAC	7 or 9 MeV	>1000 Gy/s	Not given	Gomez et al. (2022)^53^
IORT NOVAC11	5 or 7 MeV	∼4000 Gy/s	Not given	Di Martino et al. (2020)^5^
IntraOp Mobetron	6 or 9 MeV	>800 Gy/s	17.3 cm SSD	Moeckli et al. (2021)^3^
IORT NOVAC7	7 MeV	∼540 Gy/s	1.6 cm SSD	Felici et al. (2020)^54^
Eleckta Synergy	6 MeV	∼633 Gy/s	13 to 15 cm SSD	Xie et al. (2022)^55^
Varian Clinac iX	9 or 16 MeV	∼20 000 Gy/s	Internal monitor chamber	Szpala et al. (2021)^26^
Varian Clinac 21EX	16 MeV	∼2650 Gy/s	Monitor ion chamber	Poirier et al. (2021)^56^
Kinetron LINAC	4.5 MeV	60 Gy/s	Not given	Favaudon et al. (2014)^2^

First, a radiation survey was conducted for which the shielding of the pre‐existing vault was deemed sufficient for the Clinac‐FLEX system. Pre‐existing vaults that use lead shielding (as opposed to concrete) may have more difficulty in achieving the radiation safety goals due to the generation of bremsstrahlung photons. The Clinac‐FLEX system's acceptance testing and dosimetric characterization were performed using radiochromic film, optically stimulated luminescent dosimeters, and a plane‐parallel ionization chamber. The Clinac‐FLEX system could deliver a 16 MeV eFLASH beam of >1 Gy per pulse at the isocenter. It achieved a maximum dose rate of >680 Gy/s near the upper accessory mount of the linac gantry. It was observed that the 16 MeV eFLASH and CONV beam profiles were similar for applicator sizes less than 10 × 10 cm.^2^ For field sizes greater than 10 × 10 cm^2^, the eFLASH beam profile was considerably more forward‐peaked and less flat compared to the CONV beam profile. This was because the custom scattering foil used for the eFLASH beam was thinner compared to the original Varian‐designed scattering foil for the conventional 16 MeV electron beam, which prioritizes beam flatness over maximizing the dose rate.

The stability of the eFLASH beam was observed over a 4‐month interval. The most extraordinary fluctuations in output occurred during the warm‐up period, and in general, Varian's stability specification was met (i.e., the dose stability between pulses should be greater than 95%). Specifically, the dose stability was within the tolerance for 158 of the 165 measured deliveries over the initial 4‐month interval. Of the seven deliveries that deviated by more than 5%, the majority were observed during the machine warm‐up period. This is still less stable compared to a conventional clinical electron beam. However, after implementing Varian's new recommendation of turning off the PFN servo for FLASH delivery, the beam stability improved significantly (Figure 5b) and began to approach the stability of a conventional clinical linear accelerator. The PFN servo aims to ensure that the RF power sent into the accelerator cavities is optimally matched to the cavity and beam parameters. As part of this, the dose measured by the ion chamber is used as input. Since this input is no longer physically relevant in the FLASH regime, the vendor has determined that better machine performance occurs when the PFN servo is left off. Consequently, by deactivating the switch, the beam output achieves enhanced stability for the FLASH mode.

Other laboratories studying FLASH systems have observed similar results regarding dose stability.^57^ Oesterle et al.^57^ performed a short‐ and long‐term stability study of the Mobetron for the 6 MeV and 9 MeV FLASH beams. The short‐term (over 5 days) dose stability for 6 MeV and 9 MeV FLASH beams were 1.79 and 2.09%, respectively, and the long‐term (3 months) dose stability for 6 MeV and 9 MeV FLASH beams were 2.85 and 3.89%, respectively. Their research demonstrated that the beams became more stable at lower energies and shorter durations. Jaccard et al.^49^ also monitored the FLASH beam output stability for 117 days over 20 months using the Oriatron eRT6, for which the standard deviation of the output stability was 4.1%. The authors showed that the machine needed to be warmed up for 2 h prior to acquiring measurements to improve the beam stability. This procedure achieved average output stability within ± 3%, with maximum deviations of less than 10%. An Elekta Precise was also tested for FLASH beam stability based on a diode detector and GafChromic EBT3 film.^50^ A total of 20 measurements were acquired, including nine measurements during the warm‐up period (first 10 min). During the warm‐up period, the diode and radiochromic film measurements had standard deviations of 1% and 4%, respectively, whereas the respective standard deviations for the entire experiment period were 7% and 11%. Lempar et al.^50^ also showed that the FLASH beam output from the Elekta Precise decreased over time while beam stability degraded. Rahman et al.^4^ modified a Varian Clinac 2100 C/D to deliver 10 MeV eFLASH beams and performed the beam stability tests as well. Outputs during the warm‐up period were lower, for which the first measurement showed a value of 30% lower than that of the average output. Nevertheless, the beam stabilized after ten measurements.

In regards to dosimeters, no ideal dosimetry methodology currently exists for ultra‐high‐dose‐rate beams. Radiochromic film is capable of measuring high doses and dose rates, but there is a developing period preventing real‐time dosimetry.^58^ Similarly, luminescent dosimeters can be used for dosimetry in high‐dose‐rate environments but not for real‐time dosimetry.^34^ Additionally, OSLDs can only accurately measure doses up to 15 Gy.^31^, ^59^ Conventionally, the output of the linac has been monitored and controlled in real‐time through the built‐in monitor ion chambers; however, it was observed that the ion collection efficiency decreases when using the existing ion chambers for the FLASH beam.^26^, ^53^, ^58^, ^60^, ^61^, ^62^ A real‐time dosimetry monitoring system for the Clinac‐FLEX is an active area of research.

In our experiment, the plane‐parallel chamber was studied in the eFLASH environment. It was observed that the uncorrected dose‐per‐pulse curve was linear at low dose‐per‐pulse but saturated at high dose‐per‐pulse. Baghani et al.^38^ studied four different ion chambers (i.e., Semiflex, PinPoint, Advanced Markus, and Roos) in high dose‐per‐pulse conditions. They examined their recombination correction factors based on the Boag models.^39^, ^40^ The Advanced Markus chamber demonstrated the best performance compared to the other three ion chambers. While the Boag models performed well at low polarizing voltages, such as 50 V, the accuracy decreased as the polarizing voltage increased, and at 300 V, the accuracy was remarkably poor. It has been proposed by Peterson et al.^37^ that a logistic model could compensate for the disadvantage of the Boag models for ion recombination in the advanced Markus chamber, which was also highlighted in our study (Figure 6b). There are several alternative dosimetry methods being developed to resolve the challenges of ion chambers in FLASH environments, including diamond detectors, diode dosimeters, and plastic scintillators.^63^, ^64^, ^65^, ^66^

Preliminary cell studies using the Clinac‐FLEX system have demonstrated differential dose rate effects in normal and cancer breast cells (Figure 7). While the FLASH dose rate diminished the radiation‐induced morphological changes of normal breast cells, it caused similar killing of breast cancer cells than the non‐FLASH dose rate. Further research is warranted to confirm this observation. Furthermore, this experiment was limited to only comparing two dose rates: FLASH dose rate (180 Gy/s) and non‐FLASH dose rate (18 Gy/s). While the non‐FLASH dose rate was less than the perceived threshold for the FLASH effect, that is, 40 Gy/s,^11^ it was much higher than the conventional dose rate of the system (0.1 Gy/s). Although it is beyond the scope of this investigation, future studies with the FLEX system will need to systematically define the dose versus dose rate‐dependent effects of radiation on normal and cancer cells to understand the mechanism of the dose rate effect and to achieve maximum efficacy of radiation therapy for improving cancer treatment.

One limitation of the Clinac‐FLEX system is that the dose is set by the Beam Pulse Counter (i.e., the number of pulses), which is approximately 1 Gy per pulse at the isocenter. A conventional linac is able to accurately deliver dose by setting the desired number of Monitor Units (MUs). This allows for fine adjustment of the delivered dose (approximately one cGy per MU in reference conditions), whereas the Clinac‐FLEX system has a coarse adjustment of the selected delivered dose. Our laboratory is actively researching the process of adjusting the gun current of the Clinac‐FLEX system in combination with setting the desired number of pulses to select the delivered dose more finely.^50^, ^67^ However, the RF power from the klystron should be adjusted along with the gun current to ensure stable beam quality.

Similar to the conventional dose‐rate case, another limitation of the 16 MeV electron FLASH beam is the potential depth of treatment. For superficial targets, a bolus can be added to the surface of the patient's skin such that the distal end of the target is located at the therapeutic range of the electron PDD (e.g., R_90_ or R_80_). For deep‐seated targets (e.g., located beyond the practical range, R_p_) other FLASH modalities should be considered, such as FLASH with particle therapy or electrons with very high energies (VHEE).^68^

In this study, a novel system to deliver 16 MeV eFLASH was introduced, and the acceptance, commissioning, and dosimetry characterization were conducted for cell and animal research. As a means of introduction, our preliminary cell studies demonstrated the FLASH effect using the Clinac‐FLEX system, warranting further investigation. The Clinac‐FLEX system has the potential to vastly increase access to ultra‐high‐dose‐rate platforms for conducting FLASH research and further promote multi‐institutional collaborations.

## 5 CONCLUSION

The University of Nebraska Medical Center (UNMC), Faith Regional Health Services, and Varian collaborated to implement Varian's FLEX conversion for eFLASH research. This system is capable of delivering FLASH and conventional dose rates for 16 MeV electrons. Dosimetrically, the PDDs of the 16 MeV eFLASH and conventional electron beams were similar for all applicators and field sizes evaluated in this study. However, the agreement of the beam profiles diverged for large field sizes (>10 × 10 cm^2^) primarily because of the difference in the scattering foil designs. Despite there still being many studies to perform and limitations to identify before proceeding with clinical trials, the Clinac‐FLEX system has the potential to significantly increase the access to ultra‐high‐dose‐rate capabilities for scientists and clinicians and further promote multi‐institutional research on the FLASH effect.

## AUTHOR CONTRIBUTIONS

Kyuhak Oh, Kyle J. Gallagher, Ying Yan, and Su‐min Zhou contributed to the conception and design of the project, as well as conducting all experiments, corresponding data analysis, and writing, reviewing, and editing the manuscript. Megan Hyun, Diane Schott, Sarah Wisnoskie, Yu Lei, Samuel Hendley, Jeffrey Wong, and Shuo Wang contributed to a portion of the physics experiments, data analysis, and manuscript review. Brendan Graff and Christopher Jenkins contributed to a portion of the biological experiments and data analysis. Frank Rutar contributed to a portion of the safety survey and analysis. Md Ahmed, Joshua McNeur, Jeffrey Taylor, Marty Schmidt, Lasitha Senadheera, and Wendy Smith contributed to a portion of the machine acceptance test and manuscript review. Donald Umstadter and Subodh M. Lele contributed to the conception and design of the project. Ran Dai and Jianghu (James) Dong contributed to the statistical analysis. Kyuhak Oh and Kyle J. Gallagher made equal contributions to this work. Su‐min Zhou (Senior author) and Ying Yan are the corresponding authors for this work.

## CONFLICT OF INTEREST STATEMENT

University of Nebraska Medical Center is a member of the Varian FlashForward Consortium. Megan Hyun has previously received speaker honoraria from Varian Medical Systems for work unrelated to the present study.
